# Internet-Based Recruitment to a Depression Prevention Intervention: Lessons From the Mood Memos Study

**DOI:** 10.2196/jmir.2262

**Published:** 2013-02-12

**Authors:** Amy Joanna Morgan, Anthony Francis Jorm, Andrew James Mackinnon

**Affiliations:** ^1^Centre for Youth Mental HealthOrygen Youth Health Research CentreThe University of MelbourneParkvilleAustralia; ^2^Population Mental Health GroupMelbourne School of Population and Global HealthThe University of MelbourneParkvilleAustralia

**Keywords:** Internet, research subject recruitment, depressive disorder, preventive psychiatry, randomized controlled trial

## Abstract

**Background:**

Recruiting participants to randomized controlled trials of health interventions can be very difficult. Internet-based recruitment is becoming an increasingly important mode of recruitment, yet there are few detailed accounts of experiences recruiting participants to mental health interventions.

**Objective:**

To report on our experience with Internet-based recruitment to an online depression prevention intervention and pass on lessons we learned.

**Methods:**

Participants were recruited to the Mood Memos study, an online preventive depression intervention, purely through Internet-based sources. The study was targeted to adults with subthreshold depression symptoms from several English-speaking countries. A variety of online recruitment sources were trialed, including search engine advertising (Google, Yahoo!, Bing), Facebook advertising, posts in forums and online noticeboards, and promotion through relevant websites and email newsletters of mental health organizations.

**Results:**

The study website received visits from 94,808 individuals over the 14-month recruitment period. The recruitment target was reached with 1699 individuals signing up to the randomized controlled trial and 1326 fully enrolling. Most visitors arrived via Google advertising, which promoted a depression-screening questionnaire. Google advertising accounted for nearly half of the total participants who signed up to the study, at an average cost of AUD $12 per participant. Promoting the study through trustworthy organizations and websites known to participants was also effective. Recruitment techniques that were less effective were contacting forums, email groups, and community noticeboards.

**Conclusions:**

Several techniques, including Google advertising, were successful in recruiting participants to a trial evaluating an online depression intervention. Results suggest that Internet-based recruitment to mental health interventions is feasible and can be relatively affordable.

**Trial Registration:**

ACTRN12609000925246

## Introduction

It can be very difficult to meet recruitment goals in health research, particularly trials of interventions. Many studies fail to recruit the targeted number of participants or experience delays in participant recruitment [1]. A number of strategies have been proposed to improve recruitment rates [2]. Among these, Internet-based recruitment is growing in popularity, particularly in health survey research [3] and in interventions to improve physical health, such as smoking cessation interventions [4,5]. This is increasingly feasible given the high levels of access to the Internet in developed countries [6]. Internet-based recruitment has several advantages, such as the ease of reaching people from a wide geographic area including locations remote from researchers. Anonymous participation is possible, which is useful for highly sensitive topics such as illicit drug use [3] and sexuality [7]. It can also be easier to recruit large samples cost-effectively [4].

Most research on the cost of Internet-based recruitment has been published in the area of substance use and smoking cessation [4,5,8-11]. Costs per completed survey or enrolled participant have varied across (and within) these studies for a variety of reasons, such as different online advertising types (eg, Facebook, Google search, and banner advertising), the percentage of eligible participants who clicked on the ad, and the use of incentives to participate. Ramo et al [5] evaluated the cost of recruiting young adult smokers in the United States to complete an online survey about substance use through text advertisements and banner advertisements on social networking and lifestyle websites, as well as a separate study using Facebook advertising [11]. Text and banner advertisements cost an average of US $43 per completed survey, but Facebook advertising (targeted to sociodemographic characteristics and substance keywords in user profiles) cost an average of US $4.28 per completed survey. Recruitment through Google advertising was also cost-effective for several smoking cessation interventions, including 2 randomized controlled trials [4,8,9]. Google Search advertisements cost on average US $5 to $8 per registrant to QuitNet [4]. This was far less expensive than banner advertising on popular websites with broad reach, which cost up to US $476 per participant and had fewer registrants to the program. Similarly, Gordon et al [9] found an average cost per participant of US $6.70 through Google advertising, which was much less expensive than a media campaign (US $92) and newspaper advertising (US $115). Buller et al [8] found a higher average cost per participant from Google advertising (US $41), but it was the least expensive advertising type in their study, comparing favorably with US $56 for distribution of printed promotional material and US $134 for Quit-Line phone screening.

Despite the growth in Internet-based interventions for mental health problems, such as computerized cognitive behavior therapy [12], Internet-based recruitment to mental health studies is uncommon [13,14]. Evaluations of these interventions have often used traditional offline recruitment methods, such as flyers, newspaper advertising, and sourcing participants from users of health services. Recruitment problems are amplified in preventive medicine where it can be especially difficult to recruit participants to preventive trials targeting individuals at risk rather than those with a diagnosed condition (who may already be in contact with health professionals). In addition, these studies typically require large sample sizes. Trials that do not recruit participants from a health service often use media releases to advertise the study or screen members of the public through mail surveys to determine whether they meet study eligibility criteria [15].

More than a quarter of Internet users search online for information about depression, anxiety, stress, or mental health issues [16]; antidepressants are the second most searched-for treatment on WebMD [17]. Therefore, targeting individuals who use the Internet to seek information or support for mental health problems could be a useful method of recruiting participants to Internet-based mental health interventions. However, there are few detailed accounts of recruitment experiences to guide mental health researchers. Such information is critical to trial planning because recruitment success will determine the feasibility of a study, and both traditional and Internet recruitment methods can consume a substantial part of trial budgets.

We recently developed an Internet-based intervention (Mood Memos) to improve depressive symptoms in adults. The initial Mood Memos trial targeted people with mild symptoms who were at risk for depressive disorders. Thus, we did not have a geographically or service-defined population from which to sample (eg, a mental health clinic). Therefore, we decided to investigate the potential of recruiting participants solely through Internet sources. Internet-based recruitment was feasible because the intervention was completely Web-based and automated, with Web-based questionnaires used to assess eligibility. This meant that individuals who had turned to the Web for information or support about depression could seamlessly sign up to the study and start receiving the intervention immediately. Furthermore, Internet-based recruitment enabled access to a larger pool of potential participants than was possible if recruitment was restricted to our geographic location (Melbourne, Australia). This was important because the study required a large sample size to detect the predicted small effect size associated with a low-intensity preventive intervention. We explored a variety of Internet-based recruitment techniques. In this paper, we document our experience with Internet-based recruitment to a mental health intervention and the lessons we learned.

## Method

The Mood Memos study was a randomized controlled trial (ACTRN12609000925246) testing whether self-help behaviors for depression could be improved by promotional messages sent by email [18,19]. Participants received a series of automated Mood Memo emails over a 6-week period, which encouraged the use of effective self-help strategies endorsed by experts. These emails were found to reduce depression symptoms and psychological distress relative to control emails that provided information only [18]. The study was an indicated prevention trial, with a minimum of 800 participants required to have adequate power to detect the predicted small effect size. Allowing for dropouts, we set a target recruitment size of 1200 participants.

The study was open for recruitment between February 2010 and March 2011. Participants joined the study by visiting the Mood Memos website [20], and undergoing screening for subthreshold depression. There were no incentives given to participate. The website was established for the express purpose of recruiting to the study and included only the screening questionnaire, information about the study, and links for immediate help from other sources. The Patient Health Questionnaire depression scale (PHQ-9) [21] was used to screen for depression. It can be scored as a continuous measure of depression severity or by using a diagnostic algorithm to make a probable diagnosis of major depression. The frequency over the past 2 weeks of each of the 9 criterion A symptoms of a major depressive episode as defined by the *Diagnostic and Statistical Manual of Mental Disorders*, 4th Edition (DSM-IV) was assessed on a 4-point Likert scale (0=not at all; 3=nearly every day). Total scores range from 0 to 27, with cutpoints of 5, 10, 15, and 20 representing mild, moderate, moderately severe, and severe levels of depressive symptoms, respectively [22]. Because we targeted participants with subthreshold depression symptoms (clinically relevant depressive symptoms at levels that did not meet the criteria for major depression), rather than use a cut-off score, we included participants with 2 to 4 symptoms of depression [23] experienced most of the time for 2 or more weeks, which had affected work, home, or social functioning. Other inclusion criteria were aged 18 years or over; not receiving treatment for depression from a health professional (not including maintenance antidepressant medication for 6 months or more); a resident of Australia, New Zealand, the United Kingdom, Ireland, Canada, or the United States; and had access to the Internet at least weekly. Once participants were screened and found to be eligible, they provided a name and email address. A hyperlink to the baseline questionnaire package was then sent to this email address. Once the baseline assessment was complete, participants were randomized to condition and immediately sent the first of 12 Mood Memo emails. The trial was approved by the University of Melbourne Human Research Ethics Committee (HREC 0931313).

Our intention was to recruit participants from a variety of online sources, and although we initially specified some recruitment sources, we were flexible in exploring new online recruitment opportunities. We trialed a range of recruitment techniques, including search engine advertising, Facebook advertising, emails to personal and professional networks, posts in forums and online noticeboards, submitting the website to website directories, distributing an online press release, and promotion through relevant websites. Advertising costs are reported in Australian dollars (AUD); during the advertising period, AUD $1 ranged between US $0.98 and $1.02 [24].

## Results

### Overview

According to Google Analytics reports, there were 101,113 visits to the Mood Memos website from 94,808 unique visitors, accessed from 703 sources (eg, websites or direct uniform resource locator [URL] entry) over the 14-month recruitment period. The website was accessed from 118 countries, with most visiting from the United Kingdom (55.8%), Canada (15.0%), Australia (14.7%), and the United States (6.6%). The depression questionnaire screened 80,105 people; 1699 signed up to the study and 1326 completed all baseline assessments and were fully enrolled. The vast majority of website visitors came from Google advertising, which sent more than 87,000 visitors to the website. Nonpaid visitors from search engines such as Google, Bing, and Yahoo! (ie, organic search traffic) accounted for 936 visits. There were 5236 visits from other nonpaid websites, with most referrals from Psychological Research on the Net [25], Mood Disorders Society of Canada [26], StumbleUpon [27], eHealth Forum [28], and Balance NZ [29]. Direct traffic (eg, entering the website URL) accounted for 7415 visits. The results from selected attempts to promote the study online are described in more detail subsequently, and an overview of their advantages and disadvantages is shown in Table 1. Due to a technical oversight, it is not possible to provide data on the recruitment source of every participant; participants recruited through links from within emails (eg, an email newsletter) were recorded as direct visitors. Future recruitment campaigns can avoid this limitation by using Google’s URL Builder tool, which allows details about a marketing campaign to be added to the URL and then tracked by Google Analytics. Nevertheless, we can give an overview of the success of each recruitment technique and our solutions to the challenges we experienced.

**Table 1:** Overview of advantages and disadvantages of recruitment sources in the Mood Memos study.

Attributes		Recruitment source						
		Google advertising	Facebook advertising	Forums	Links on other websites	Online noticeboards^a^	Google or Yahoo groups	Emails from relevant organizations
**Advantages**								
	Broad reach	✓	✓					
	Targeting	✓	✓	✓			✓	
	Low ongoing effort	✓	✓					
	No cost			✓	✓	✓	✓	✓
	High conversion rate							✓
	Implicit endorsement				✓			✓
**Disadvantages**								
	Narrow reach						✓	✓
	High learning curve	✓	✓					
	High cost	✓	✓					
	Low conversion rate	✓						
	Time-consuming			✓			✓	
	Difficulty acquiring permission			✓	✓	✓	✓	✓

^a^ For example, Craigslist and Gumtree.

### Google Advertising

Search engine advertising was trialed with Google, Yahoo!, and Bing between October 2010 and March 2011 (Bing and Yahoo! merged their advertising services during the trial). Resources were directed toward Google advertising because it proved more effective than either Yahoo! or Bing. Search engine advertising services display a short advertisement when a user searches for certain keywords, which are preselected by the advertiser. Advertisers competitively bid on specific keywords to get their ads shown. The number of times an ad is displayed is dependent on the budget of the advertiser, competition from other advertisers for the chosen keywords, and the quality of the ad. Google provides advice on how to improve ad quality, and there are numerous books and websites that offer similar advice.

**Figure 1:**
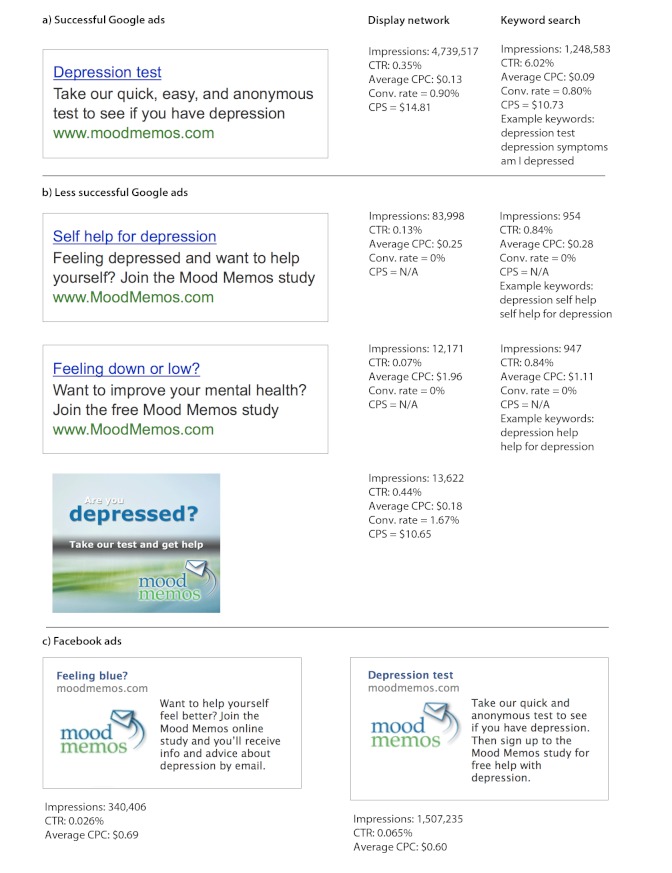
Examples of paid advertisements in the Mood Memos study. CTR=click-through rate (percentage of clicks per impressions); CPC=cost per click; Conv rate=conversion rate (percentage of sign-ups per clicks); CPS=cost per sign-up; N/A=not applicable.

**Figure 2:**
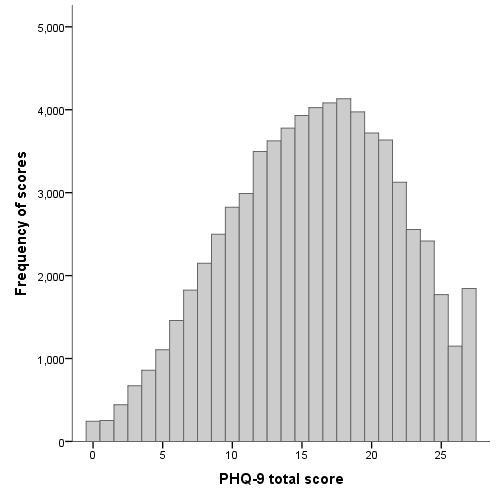
Distribution of scores on the Patient Health Questionnaire (PHQ-9) from those participants screened through Google advertising. Subthreshold depression scores for participants eligible for inclusion in the Mood Memos study ranged from 3 to 17 on the PHQ-9.

Advertisers can choose to pay each time an ad is displayed or each time it is clicked on (pay-per-click). We chose pay-per-click because this is more cost-effective for ads whose primary purpose is to direct a viewer to a website rather than promote a brand. There are a variety of advertising settings that can be adjusted and experimented with, including daily budget, targeted locations, ad scheduling for time of day or day of the week, and keyword search term precision. We experimented with a number of different keywords and ads, and found that the most successful combination were ads that targeted individuals seeking a depression test or information about symptoms because they thought they might have depression (see Figure 1 for examples of Google advertisements used in the study). The depression test ad and keyword combination attracted a large number of searches, a reasonable percentage of clicks per display of the ad (an average click-through rate of 6.0%) [30], but was still affordable (AUD $0.08 per click). The average cost per participant sign-up using this keyword was AUD $9.86. Although each click on this ad was inexpensive, less than 1% of clicks led to a participant signing up to our study. This low conversion rate was not unexpected, given that participation was restricted to those with a narrow range of depressive symptoms, and because Google users did not necessarily visit the Mood Memos website to sign up to the study, but rather to find out if they were depressed. In fact, most individuals who visited the website through Google advertising returned a screening result of probable major depression (5 or more depressive symptoms) rather than subthreshold depression (63.7% versus 20.7%, respectively). Figure 2 shows the distribution of scores on the PHQ-9 from those who were screened via Google advertising. Other recruitment sources returned a lower rate of probable major depression (49.4%) than Google advertising.

Keywords and ads related to self-help or coping with depression were also used (eg, coping with depression, depression self-help, and how to deal with depression). However, although they were more specific to the study, they attracted fewer searches, a lower click-through rate, and consequently fewer participants. Either we did not hit upon appropriate keywords or our ads were not attractive to users searching with these keywords.

Keyword bids were more expensive initially, but reduced in price as more people clicked on our ads and our quality score improved. The quality score is an index calculated by Google based on an estimate of the relevancy and usefulness of the ad and Web page to which it leads [31]. In our case, the “depression test” keyword cost AUD $2.74 per click initially, but within 10 days had reduced to AUD $0.14. The cost per click will vary with the targeted geographic location because it depends on how many other advertisers are also targeting particular keywords in that location. Although we targeted all 6 countries equally, our ads were displayed less frequently in the United States and more often in the United Kingdom. This was because we chose automatic bidding for keywords, which meant that because of greater competition in the United States for our keywords, sometimes we were outbid by other advertisers or our ads were not ranked highly enough to be displayed in the first page of search results.

Overall, our ads were shown 1,251,262 times and received 75,225 clicks from Google keyword search. The average click-through rate was 6.01%, the average cost per click was AUD $0.09, and the average sign-up rate from those who clicked on the ads was 0.80%. This led to 602 people signing up to the study, at an average cost of $10.75 per person.

Display network advertising on Google was also utilized. This shows advertisements (text and other media) on websites that have been contracted to display advertising from Google. Specific websites can be targeted or Google can select appropriate websites based on the keywords chosen by the advertiser. Google display network advertising was chosen as an alternative to banner advertising on popular websites, which is prohibitively expensive. Our ads were displayed 4,759,393 times and received 16,883 clicks. The majority of clicks were produced from About.com Depression [32] and NetDoctor [33]. Our click-through rate was lower than for keyword search advertising (0.35%), as found by other researchers [3]. It was also slightly more expensive than search engine advertising, costing an average of AUD $0.13 per click over the life of the campaign, but it had a slightly higher participant sign-up rate (0.91%) compared to search engine advertising (0.80%). Display network advertising led to 153 people signing up to the study at an average cost of AUD $14.71 per sign-up.

Overall, advertising on Google was an effective recruitment source. Of 1699 participants who initially signed up to the study, 755 (44.44%) were recruited through Google advertising. These participants were recruited over a 6-month period at an average cost of AUD $11.55 per participant.

### Facebook

We tested advertising on Facebook, but found it was less cost-effective than advertising on Google, so we did not persist with it. Advertising on Facebook works differently than search engine advertising because ads are targeted to specified demographics (eg, age, gender, location, relationship status, and education) and user interests rather than search keywords. This was less useful for the Mood Memos study, which was not targeting narrowly defined demographic subgroups, other than adults 18 years or over from the 6 eligible countries (Australia, New Zealand, the United Kingdom, Ireland, Canada, or the United States). However, we experimented by targeting our ads to people whose demographics or likes/interests indicated they were at higher risk of depression (eg, females, “depressed,” “lonely,” and “unemployed”). Our ads were displayed 2,051,216 times, received 1115 clicks (click-through rate 0.05%), and cost an average of AUD $0.62 per click. Facebook advertising recruited 35 participants, averaging $19.89 per participant. Figure 1 shows examples of the Facebook advertisements used in the study. Other studies with focused participant demographics have had more success in recruiting participants through Facebook [34].

### Forums

Online depression forums (or Internet support groups) are very common and many have tens of thousands of members [35]. We thought mental health forums would be a good recruitment source of people experiencing depression symptoms who would be interested in participating in the study. Some forums also have sections dedicated to research studies seeking participants. We performed searches on Google and Bing to identify forums in which to post, as well as following links from other websites. However, this recruitment source was much less useful than expected. We approached 58 forums related to depression and other related problems, but only 25 responded with permission to post about the study. Many of the larger forums had policies that outright denied permission to post about research studies. Although some smaller forums gave permission to post, this was usually a time-consuming process involving signing up to the forum and creating a user account, then identifying the forum moderator or administrator and contacting them for permission to post, and then finally submitting the post and monitoring responses. We found recruitment through online forums of limited use, possibly because many of the users of these forums were not eligible to participate because they were already too depressed or were receiving treatment from a professional. Furthermore, other studies that have had success recruiting through online forums have been for cross-sectional questionnaires rather than longitudinal interventions [36]. Perhaps the lack of response to our forum recruitment drive may have been partly because of the additional obligations required for participation in a longitudinal trial.

### Links From Websites

A variety of mental health websites were contacted, informed about the Mood Memos study, and asked to promote it on their website or via email newsletters. Many websites would only agree if there was a reciprocal link on the Mood Memos website. A new page of supporters was created to accommodate this requirement. This page thanked each organization or website that had helped promote the study to participants. Some websites were generous and included a link and blurb on their home page; others listed the website within a section of their site that contained links to other interesting websites. The Mood Memos website was also listed on several websites that promote participation in research in general or psychology in particular. The advantage of promoting the study through other mental health websites was the broad exposure to the Mood Memos website offered by these credible, well-established websites that had hundreds or thousands of daily visitors.

### Online Community Noticeboards

We explored the effectiveness of posting an invitation to participate in the study on websites that function as online community noticeboards (eg, Craigslist [37] and Gumtree [38]). It is free to post a classified ad on these websites, and other survey-based studies have found them an effective recruitment source [5]. However, although free, these websites are designed to offer products and services to local residents only. Posting in multiple categories or locations concurrently (eg, Melbourne and Sydney) is not allowed, and the American and Canadian Craigslist websites required a local mobile phone number to confirm the advertisement, preventing advertisements by foreigners. On Craigslist, we posted a study announcement in 4 cities (Melbourne, Sydney, London, and Birmingham) in various categories (volunteers and therapeutic services), but had only 29 visits, with 3 participants signing up to the study. Similarly, we posted information about the study in 4 cities with Gumtree Australia, United Kingdom, United States, and Ireland, but had only 1 participant sign up via these ads. We had more success posting a notice in the online student noticeboard at the University of Melbourne (where the study researchers were based). This website is a portal of official services (eg, subject timetables) for students at the University of Melbourne, but also allows moderated notices to be posted that may be of interest to students. We posted the study notice 4 times over the recruitment period, and observed a noticeable increase in visits and enrollments coinciding with each posting. This success may be because the notice was targeted at a group at high risk for depression (ie, university students [39]) and because students may have trusted the study because it was conducted by staff members of their university.

### Email Groups or Lists

We also tried contacting members of various email groups or lists. Yahoo! and Google provide a free service in which individuals with shared interests can join an online group and share messages and information. These messages are sent to email accounts or can be viewed in a Web browser. There are hundreds of groups related to mental health conditions or risk factors, but many of these have few members or have been overtaken by spam messages. We contacted 103 relevant groups with a reasonable number of members to advertise the study and 32 gave their permission. Again, this was a time-consuming process because groups often had to be joined before the owner of the group could be contacted for permission to post to the rest of the group. Other email lists that were more successful in recruiting participants were not Yahoo! or Google groups, but rather the email lists of mental health organizations, such as beyondblue (the Australian national depression initiative) [40] and Mental Health First Aid (MHFA) [41]. Promotion through the networks of these organizations led to a significant spike in visitors to the Mood Memos website and enrollments in the study.

## Discussion

The Mood Memos study demonstrated that it is possible to recruit a large sample to a randomized controlled trial of a mental health intervention purely through Internet-based sources. We were able to meet our ambitious recruitment target over a period that was only marginally longer than planned. Participants were recruited largely through a combination of Google advertising and promotion via the online networks of mental health organizations and websites. Recruiting participants to randomized controlled trials can be very difficult, and many trials do not reach their target sample size [2]. Online recruitment can potentially reach a wider pool of potential participants more cost-effectively than traditional techniques, such as media advertising. However, promoting the study online was not without difficulty. Google advertising involved a steep learning curve and much trial and error in working out the optimal combination of ads and keywords that competed well against other advertisers, were affordable, and were searched for frequently enough to be worthwhile. Researchers using Google should be aware that establishing a campaign is not sufficient for success, and that monitoring performance during the recruitment period is critical. Changes and refinements will almost certainly be needed in the course of a recruitment campaign. In comparison to Google, many other recruitment techniques, although free, were time-consuming and less effective.

Despite recruiting a large sample, recruitment was constrained by a number of factors, including the nature of the intervention, trial design, and eligibility criteria. For example, some recommended techniques in website promotion, such as search engine optimization, had limited application for the Mood Memos website. Search engine optimization involves designing websites so that users can find them easily through search engines such as Google. Google often updates its algorithms that determine website rankings in search results. Current recommendations by experts in the field are to provide quality content, rather than rely on many incoming links from other websites [42]. Because the Mood Memos website was designed to have minimal content (just the depression-screening questionnaire, information about the study, and links to resources for more help), other techniques to reach Internet users were essential. The choice of control intervention may also have increased the difficulty of recruiting participants to the study. To keep participants blind to which condition was the control, the promotion of the study could not solely focus on self-help or coping with depression, although this would probably have generated more interest from potential participants. Using a wait-list control may have made recruitment easier because the study could have been promoted as a way of learning techniques to cope with depressive symptoms. Instead, the study was promoted in general terms as a way of receiving expert information and advice about depression. Furthermore, targeting people at risk of major depression was difficult. Experience in the Netherlands has shown that there is minimal uptake of indicated preventive interventions, even when they are available at little or no cost [43]. Part of the reason for this may be that individuals may lack self-awareness or have insufficient mental health literacy to understand that their distress could be an early sign of depression. Much of the delay in seeking professional help for depression is due to a lack of problem recognition [44]. The difficulty of recruiting participants to the Mood Memos study from websites, forums, and email groups dedicated to mental health problems may have been because users of these had identified that they had depression, but their symptoms were too severe to meet admission criteria. This may have been why Google advertising targeting people who thought they might be depressed was a superior method of recruiting participants to the study. Unfortunately, Google advertising is not free, but an advantage is that it can occur in the background; it needs minimal attention once properly established.

Our experience also highlighted the importance of undertaking formal usability tests of the website sign-up process. Although we tested it informally, after launching the website we discovered that some participants could not find the sign-up button because it was below the plain language statement. Our experience indicates that there may be tension between best practice for Web usability and the current practice of obtaining informed consent by providing a long and detailed text-based information sheet to participants. Over the years, printed information sheets have increased in length, becoming more comprehensive to meet ethics requirements. Yet this increase in completeness sacrifices comprehension because longer forms are less likely to be read and understood [45]. These issues are compounded when information sheets are presented online because less text is read online compared to print, and users may miss information or instructions on how to participate if they have to scroll down to view it [46]. These issues are worth considering to avoid wasting effort attracting visitors to your website only to lose them during the sign-up process.

Internet-based recruitment does have limitations. It was particularly suitable for our study because we did not require any face-to-face assessments of participants during the study. This may not be feasible for many evaluations of mental health interventions, even when Internet-based, because diagnostic psychiatric interviews are usually preferred to self-report assessments. However, other researchers have combined Internet-based recruitment with face-to-face assessments in their research [34]. Internet-based recruitment may also lead to low rates of participation from groups that are less likely to use the Internet, such as older adults, the less educated, and those with low incomes [47]. However, this does not necessarily need to be the case because reaching samples that are more representative is possible through demographic targeting with Facebook [34]. In addition, certain kinds of Internet advertising may lead to greater participation from groups less likely to participate through traditional recruitment techniques (eg, men and ethnic minorities) [4].

Combining Internet-based recruitment with Internet-based assessment poses challenges to research integrity, including the potential for multiple, fraudulent enrollments. This can be problematic if there are incentives to participate [48]. Although we found Internet-based recruitment effective in reaching individuals with subthreshold depression who wanted to improve their mental health, others have found it failed to fulfill its promise [49] and is often more challenging than first anticipated [7,50]. There is limited knowledge about which factors contribute to successful Internet-based recruitment, but it may be important to use techniques that build rapport and gain the trust of potential participants, and to balance broad exposure with appropriate targeting and tailoring of recruitment messages [3]. If direct rapport is not feasible, then derived rapport may be sufficient. This can be achieved by recruiting participants through trusted people or organizations that have an existing link with potential participants. In our study, the effectiveness of recruitment through the University of Melbourne, beyondblue, and MHFA may indicate the importance of this factor.

Internet-based recruitment is becoming an increasingly important mode of recruitment; however, there are limited data available to guide researchers on the best recruitment strategies. Our study showed that several techniques, including Google advertising, were successful in recruiting participants to an online depression intervention. Our results suggest that Internet-based recruitment to mental health interventions is feasible and can be relatively affordable.

## Abbreviations

AUD: Australian dollars
MHFA: Mental Health First Aid
NHMRC: National Health and Medical Research Council
PHQ-9: Patient Health Questionnaire depression scale
URL: uniform resource locator

## References

[1] Campbell MK, Snowdon C, Francis D, Elbourne D, McDonald AM, Knight R, Entwistle V, Garcia J, Roberts I, Grant A, Grant A, STEPS group (2007). Recruitment to randomised trials: strategies for trial enrollment and participation study. The STEPS study. Health Technol Assess.

[2] Treweek S, Pitkethly M, Cook J, Kjeldstrøm M, Taskila T, Johansen M, Sullivan F, Wilson S, Jackson C, Jones R, Mitchell E (2010). Strategies to improve recruitment to randomised controlled trials. Cochrane Database Syst Rev.

[3] Temple EC, Brown RF (2011). A comparison of internet-based participant recruitment methods: Experiences engaging the hidden population of cannabis users in research. Journal of Research Practice.

[4] Graham AL, Milner P, Saul JE, Pfaff L (2008). Online advertising as a public health and recruitment tool: comparison of different media campaigns to increase demand for smoking cessation interventions. J Med Internet Res.

[5] Ramo DE, Hall SM, Prochaska JJ (2010). Reaching young adult smokers through the internet: comparison of three recruitment mechanisms. Nicotine Tob Res.

[6] (2011). Australian Bureau of Statistics.

[7] Alessi EJ, Martin JI (2010). Conducting an internet-based survey: Benefits, pitfalls, and lessons learned. Soc Work Res.

[8] Buller DB, Meenan R, Severson H, Halperin A, Edwards E, Magnusson B (2012). Comparison of 4 recruiting strategies in a smoking cessation trial. Am J Health Behav.

[9] Gordon JS, Akers L, Severson HH, Danaher BG, Boles SM (2006). Successful participant recruitment strategies for an online smokeless tobacco cessation program. Nicotine Tob Res.

[10] Graham AL, Fang Y, Moreno JL, Streiff SL, Villegas J, Muñoz RF, Tercyak KP, Mandelblatt JS, Vallone DM (2012). Online advertising to reach and recruit Latino smokers to an internet cessation program: impact and costs. J Med Internet Res.

[11] Ramo DE, Prochaska JJ (2012). Broad reach and targeted recruitment using Facebook for an online survey of young adult substance use. J Med Internet Res.

[12] Andrews G, Cuijpers P, Craske MG, McEvoy P, Titov N (2010). Computer therapy for the anxiety and depressive disorders is effective, acceptable and practical health care: a meta-analysis. PLoS One.

[13] Meyer B, Berger T, Caspar F, Beevers CG, Andersson G, Weiss M (2009). Effectiveness of a novel integrative online treatment for depression (Deprexis): randomized controlled trial. J Med Internet Res.

[14] Christensen H, Griffiths KM, Mackinnon AJ, Brittliffe K (2006). Online randomized controlled trial of brief and full cognitive behaviour therapy for depression. Psychol Med.

[15] Christensen H, Griffiths KM, Jorm AF (2004). Delivering interventions for depression by using the internet: randomised controlled trial. BMJ.

[16] Fox S, Jones S (2009). The social life of health information: Americans' pursuit of health takes place within a widening network of both onlineoffline sources.

[17] Fox S (2011). Health topics: 80% of internet users look for health information online.

[18] Morgan AJ, Jorm AF, Mackinnon AJ (2012). Email-based promotion of self-help for subthreshold depression: Mood Memos randomised controlled trial. Br J Psychiatry.

[19] Morgan AJ, Jorm AF, Mackinnon AJ (2011). Protocol for a randomised controlled trial investigating self-help email messages for sub-threshold depression: the Mood Memos study. Trials.

[20] Mood Memos.

[21] Kroenke K, Spitzer RL, Williams JB (2001). The PHQ-9: validity of a brief depression severity measure. J Gen Intern Med.

[22] Kroenke K, Spitzer RL, Williams JB, Löwe B (2010). The Patient Health Questionnaire Somatic, Anxiety, and Depressive Symptom Scales: a systematic review. Gen Hosp Psychiatry.

[23] American Psychiatric Association (1994). Diagnostic and Statistical Manual of Mental Disorders: DSM-IV.

[24] OANDA Corporation.

[25] Psychological Research on the Net.

[26] Mood Disorders Society of Canada.

[27] StumbleUpon.

[28] eHealth Forum.

[29] Balance NZ.

[30] Gabbert E (2010). WordStream.

[31] Google AdWords.

[32] About.com.

[33] NetDoctor.

[34] Fenner Y, Garland SM, Moore EE, Jayasinghe Y, Fletcher A, Tabrizi SN, Gunasekaran B, Wark JD (2012). Web-based recruiting for health research using a social networking site: an exploratory study. J Med Internet Res.

[35] Griffiths KM, Calear AL, Banfield M, Tam A (2009). Systematic review on Internet Support Groups (ISGs) and depression (2): What is known about depression ISGs?. J Med Internet Res.

[36] Ip EJ, Barnett MJ, Tenerowicz MJ, Perry PJ (2010). The touro 12-step: a systematic guide to optimizing survey research with online discussion boards. J Med Internet Res.

[37] Craigslist.

[38] Gumtree.

[39] Stallman HM (2010). Psychological distress in university students: A comparison with general population data. Aust Psychol.

[40] beyondblue: the national depression initiative.

[41] Mental Health First Aid Australia.

[42] Ramos A, Cota S (2009). Search Engine Marketing.

[43] Cuijpers P, van Straten A, Warmerdam L, van Rooy MJ (2010). Recruiting participants for interventions to prevent the onset of depressive disorders: possible ways to increase participation rates. BMC Health Serv Res.

[44] Thompson A, Issakidis C, Hunt C (2008). Delay to Seek Treatment for Anxiety and Mood Disorders in an Australian Clinical Sample. Behaviour Change.

[45] Beardsley E, Jefford M, Mileshkin L (2007). Longer consent forms for clinical trials compromise patient understanding: so why are they lengthening?. J Clin Oncol.

[46] Krug S (2000). Don't Make Me Think!: A Common Sense Approach to Web Usability.

[47] Zickuhr K, Smith A (2012). Digital differences.

[48] Bowen AM, Daniel CM, Williams ML, Baird GL (2008). Identifying multiple submissions in Internet research: preserving data integrity. AIDS Behav.

[49] Koo M, Skinner H (2005). Challenges of internet recruitment: a case study with disappointing results. J Med Internet Res.

[50] Benfield JA, Szlemko WJ (2006). Internet-based data collection: promises and realities. Journal of Research Practice.

